# *hMSH2* is the most commonly mutated MMR gene in a cohort of Greek HNPCC patients

**DOI:** 10.1038/sj.bjc.6602260

**Published:** 2005-01-11

**Authors:** A Apessos, M Mihalatos, I Danielidis, G Kallimanis, N J Agnantis, J K Triantafillidis, G Fountzilas, P A Kosmidis, E Razis, V A Georgoulias, HeCOG, HeHeGI, G Nasioulas

**Affiliations:** 1 Molecular Biology Research Center HYGEIA – ‘Antonis Papayiannis’, Kifissias Ave & Erythrou Stavrou 4 Str, 15123 Maroussi, Athens, Greece; 2 Gastroenterology Department, DTCA HYGEIA, Athens, Greece; 3 Department of Pathology, Medical School, University of Ioannina, Greece; 4 Department of Gastroenterology, Peripheral General Hospital of Nikaia, Greece; 5 Aristotle University of Thessaloniki, Greece; 6 2nd Pathology – Oncology Clinic, DTCA HYGEIA, Athens, Greece; 7 1st Pathology – Oncology Clinic, DTCA HYGEIA, Athens, Greece; 8 Medical School of the University of Crete, Heraklion, Crete, Greece

**Keywords:** HNPCC, *hMLH1*, *hMSH2*, Greece, rearrangements, MLPA

## Abstract

Germline mutations in genes encoding proteins involved in DNA mismatch repair are responsible for the autosomal dominantly inherited cancer predisposition syndrome hereditary nonpolyposis colorectal cancer (HNPCC). We describe here analysis of *hMLH1* and *hMSH2* in nine Greek families referred to our centre for HNPCC. A unique disease-causing mutation has been identified in seven out of nine (78%) families. The types of mutations identified are nonsense (five out of seven) (*hMLH1*: E557X, R226X; *hMSH2*: Q158X, R359X and R711X), a 2 bp deletion (*hMSH2* 1704_1705delAG) and a 2.2 kb *Alu*-mediated deletion encompassing exon 3 of the *hMSH2* gene. The majority of mutations identified in this cohort are found in *hMSH2* (77.7%). Furthermore, four of the mutations identified are novel. Finally, a number of novel benign variations were observed in both genes. This is the first report of HNPCC analysis in the Greek population, further underscoring the differences observed in the various geographic populations.

Hereditary nonpolyposis colorectal cancer (HNPCC) is the most common inherited syndrome predisposing to colorectal cancer (CRC), accounting for 5–10% of the total CRC (Lynch and de la Chapelle, 1999; MIM 114500). Hereditary nonpolyposis colorectal cancer is characterised by early-onset CRC (mean age at diagnosis ∼45 years) and an increased incidence of cancer in other organs such as the endometrium, stomach, small bowel, ovary, hepatobiliary tract, renal pelvis and ureter (Watson and Lynch, 1993; Lynch and de la Chapelle 1999).

Hereditary nonpolyposis colorectal cancer segregates in an autosomal dominant manner and it is caused by germline mutations in a group of genes encoding proteins involved in the DNA mismatch repair (MMR) pathway. At least five genes of the pathway, namely *hMLH1, hMSH2, hMSH6, hPMS1* and *hPMS2*, have been implicated in HNPCC (Leach *et al*, 1993; Papadopoulos and Lindblom, 1997; Peltomäki and Vasen, 1997). However, the majority of mutations (∼90%) have been identified in *hMLH1* (∼50%) and *hMSH2* (∼40%). Germline mutations that have been identified in *hMLH1* and *hMSH2* are scattered throughout the coding regions of the two genes with no obvious phenotype–genotype correlation. The majority of mutations identified are small insertions/deletions leading to frameshifts and truncated protein products. In addition, a large number of nonsense mutations have been identified (Peltomäki and Vasen, 1997). In recent years, an increasing number of reports have shown that ∼10–30% of mutations responsible for HNPCC are large genomic rearrangements affecting one or more exons of the two genes (Wijnen *et al*, 1998; Gille *et al*, 2002; Wagner *et al*, 2003). This, in addition to the genetic heterogeneity and clinical variability among HNPCC families, makes identification of germline mutations in families suspected to suffer by HNPCC laborious and time consuming. Use of tissue microarray immunohistochemistry expression analysis can help pinpoint the affected gene thereby targeting mutation screening directly to that gene, cutting down on cost and time of analysis (Hendriks *et al*, 2003; Jourdan *et al*, 2003; Hardisson *et al*, 2003). However, characterisation of the disease causing mutation in afflicted families is essential as it allows identification of carrier relatives, who may require appropriate surveillance and alleviates noncarrier individuals from costly and intrusive surveillance.

## MATERIALS AND METHODS

### Patients

To date, 64 individuals from 28 Greek families with CRC were referred to our centre through the Oncology and Gastroenterology Departments of the Diagnostic and Therapeutic Center of Athens HYGEIA and other hospitals throughout Greece. Following an interview with as many family members as possible, a detailed family tree is constructed. This is then reviewed by three independent scientists in the laboratory in order to make a decision on the risk of the family to be affected by HNPCC. Based on the Amsterdam II (Vasen *et al*, 1999) and/or Bethesda Criteria (Rodriguez-Bigas *et al*, 1997), nine of the 28 families were selected for molecular analysis of *hMLH1* and *hMSH2*. The study population, therefore, consisted of seven Amsterdam positive families, one Bethesda positive and one family who did not meet the criteria but were included due to the clustering of CRC.

Screening has been completed in 30 individuals from nine families. Testing was initially carried out on DNA from an affected family member and upon detection of an inactivating mutation the rest of the family members were directly tested for this mutation.

Due to the large proportion of genomic rearrangements that have been shown to be responsible for HNPCC, analysis is initially carried out using multiplex ligation-dependent probe amplification (MLPA) for screening for such mutations in *hMLH1* and *hMSH2*. If a genomic rearrangement is not detected then PCR for all exons and splice junctions of the two genes is carried out. Screening for point mutations and small insertions/deletions is carried out in the majority of amplicons using denaturing high-performance liquid chromatography (dHPLC). Denaturing high-performance liquid chromatography is not carried out for exons containing polymorphic repeats, that is, *hMLH1* exon 12 and *hMSH2* exons 2 and 5. These three exons and all those indicated by an abnormal dHPLC elution profile are subjected to cycle sequencing.

### DNA and RNA isolation

Genomic DNA and RNA were purified from peripheral blood leukocytes or tissue using standard extraction protocols.

### Multiplex ligation-dependent probe amplification (MLPA)

Multiplex ligation-dependent probe amplification was carried out using the P003 MSH2/MLH1 kit (MRC-Holland, Netherlands) as instructed by the manufacturer. Fragment analysis was carried out on the ABI Prism® 310 Genetic Analyzer using TAMRA-500 as size standard. A peak pattern of 42 peaks ranging in size from 130 to 472 nt is obtained (Gille *et al*, 2002).

### PCR amplification

The complete coding sequence of *hMLH1* and *hMSH2* including splice junctions was amplified by PCR. Primers used have been described by others (Holinski-Feder *et al*, 2001). Reactions of 50 *μ*l were heated on a PTC-200 MJ Research Thermocycler (MJ Research Inc, USA) at 95°C for 5 min then cycled 35 times of denaturation at 95°C for 40 s, annealing at the appropriate temperature for 30 s and extension at 72°C for 30–60 s, followed by a final extension step at 72°C for 6 min. Reaction mixture was 20 mM Tris HCl (pH 8.4), 50 mM KCl, 1.5 mM Mg^2+^, 200 *μ*M each dNTP, 1.5 U Taq DNA polymerase (Invitrogen, UK) or 2.5 U Optimase polymerase (Transgenomic) and 15 pmol of each primer.

### Denaturing high-performance liquid chromatography analysis

The WAVE DNA Fragment Analysis System (Transgenomic, Inc, USA) and associated WAVE-Maker™ software were used as previously described (Mihalatos *et al*, 2003a).

### Sequence analysis

Purification of the PCR products was performed using the Concert Rapid PCR purification or gel extraction system kits (Marligen Biosciences Inc, USA). Automated cycle sequencing for both strands was performed with the ABI Prism® 310 Genetic Analyzer using the BigDye Terminator Cycle Sequencing kit. Sequences obtained were aligned, using Sequencher® PC software (Gene Codes, USA), with normal sequences from Genbank and ENSEMBL (*hMLH1*: AY217549; *hMSH2*: ENSEMBL ENST00000233146) and examined for the presence of mutations. All nucleotide numbers refer to the wild-type cDNA.

### Long PCR

The deletion in *hMSH2* exon 3 was confirmed by long PCR using the Expand High Fidelity PCR System (Roche, Germany) according to the manufacturer's instructions. PCR products were separated by agarose gel electrophoresis and visualised by EtBr staining.

### RT–PCR

Total RNA was extracted from peripheral blood leukocytes of patients from family D using Trizol (Life Technologies, UK), according to the manufacturer's instructions. First-strand synthesis was performed by denaturing approximately 500–1000 ng total RNA and random hexamers (5 *μ*M final concentration) for 4 min at 70°C, followed by snap freezing on ice and addition of dNTPs (0.5 mM final concentration), 1 U *μ*l^−1^ recombinant RNase inhibitor (Invitrogen, UK) and 200 U MMLV reverse transcriptase (Invitrogen, UK). The mixture was incubated at 37°C for 1 h followed by denaturation of the enzymes at 95°C for 5 min. In total, 4 *μ*l of cDNA were used for subsequent PCR amplification.

## RESULTS

To date, 30 individuals from nine Greek families at high risk of having HNPCC, have been screened to our centre for genetic testing. Of these, MSI analysis was carried out in six patients for whom matching normal and tumour tissue was available.

Our strategy is based on an initial screening of genomic DNA for large genomic rearrangements of *hMLH1* and *hMSH2* using the recently described method MLPA (Schouten *et al*, 2002). If a rearrangement is not detected, PCR amplification of all exons and splice junctions of the two genes is carried out, using primers previously described by others (Holinski-Feder *et al*, 2001). For the majority of amplicons, dHPLC is used as a mutation screening test, followed by direct sequencing for characterisation of mutations indicated by dHPLC. Denaturing high-performance liquid chromatography was not carried out for amplicons containing repetitive polymorphic sequences, that is, exon 12 of *hMLH1* and exons 2 and 5 of *hMSH2.*

In seven families (3335, 5838, 7562, 8344, 8902, 9663 and 10107 in Table 1), seven different mutations have been identified. Four of the mutations are novel nonsense mutations in the *hMLH1* and *hMSH2* genes. One family (7562 in Table 1) carries a 2.2 kb deletion encompassing exon 3 of the *hMSH2* gene while the remaining two identified mutations have already been described in the ICG-HNPCC database (http://www.nfdht.nl (ICG-HNPCC mutation database).

### Families with novel mutations

**Figure 1:**
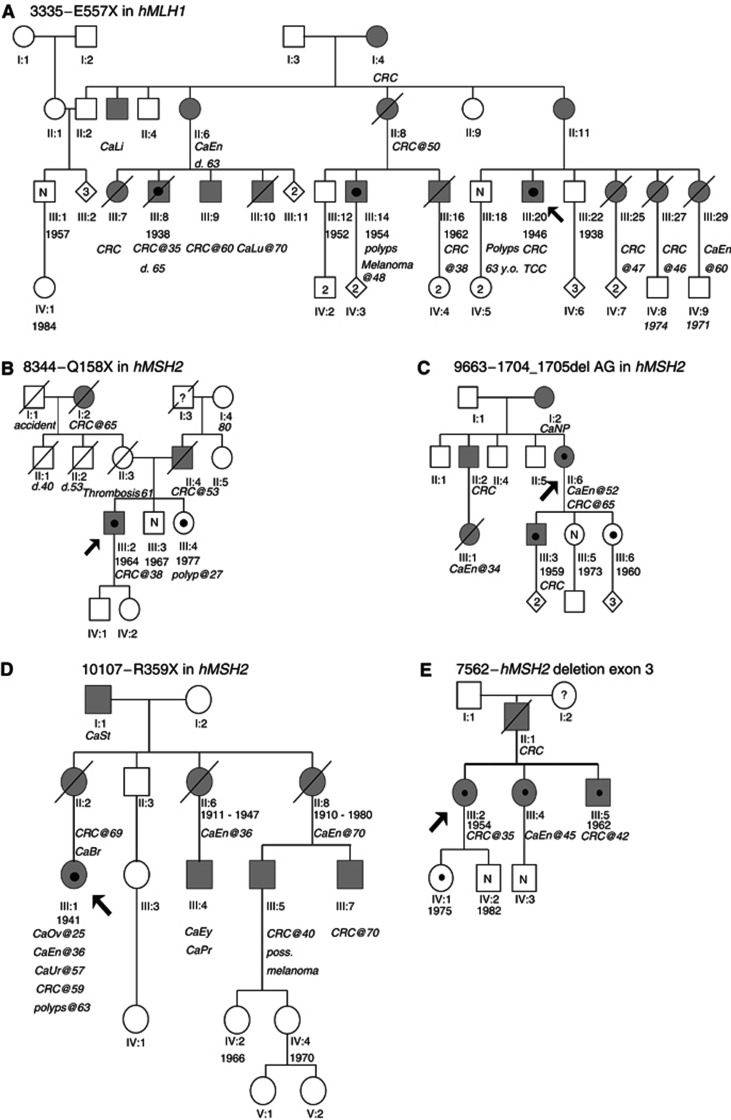

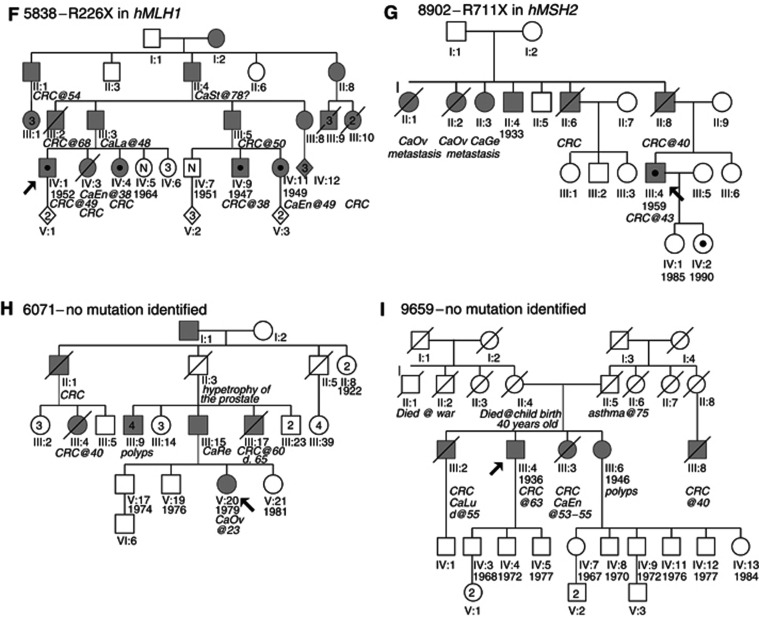
Pedigrees of families investigated for germline mutations of the *hMLH1* and *hMSH2* genes. Grey symbols indicate individuals with cancer. The proband is indicated by an arrow. A black circle inside a symbol indicates individuals found to carry the mutation. N=individual tested for the family mutation and was found to be of normal genotype. CRC=colorectal cancer, CaEn=endometrial cancer, CaLi=liver cancer, CaLu=lung cancer, TCC=transitional cell carcinoma, CaNP=nasopheryngeal cancer, CaSt=stomach cancer, CaOv=ovarian cancer, CaEy=cancer of the eye, CaPr=prostate cancer, poss.=possibly, CaLa=cancer of the larynx, CaGe=‘gynaecological’ cancer, CaRe=cancer of the rectum.

In family 3335 (Figure 1A and Table 1), the identified mutation is a single base substitution in exon 15 of the *hMLH1* gene. The mutation, 1669G>T (Figure 3A), converts the glutamine at codon 557 to a STOP codon. The mutation was originally identified in a patient (III:20,Figure 1A and 2A) who had transitional cell carcinoma and CRC. Tumours of this patient were found to be MSI positive. MSI analysis of polyps resected from the patient's brother (III:18 Figure 1A) was negative. Individual III:18 has not developed a malignancy until the age of 65. Mutation analysis of *hMLH1* exon 15 revealed that he did not carry the mutation identified in his brother. The mutation was however identified in two more patients of this family (III:14 and III:8, Figure 1A). In the first patient a malignant melanoma had been diagnosed while the latter suffered from CRC.

**Figure 2:**
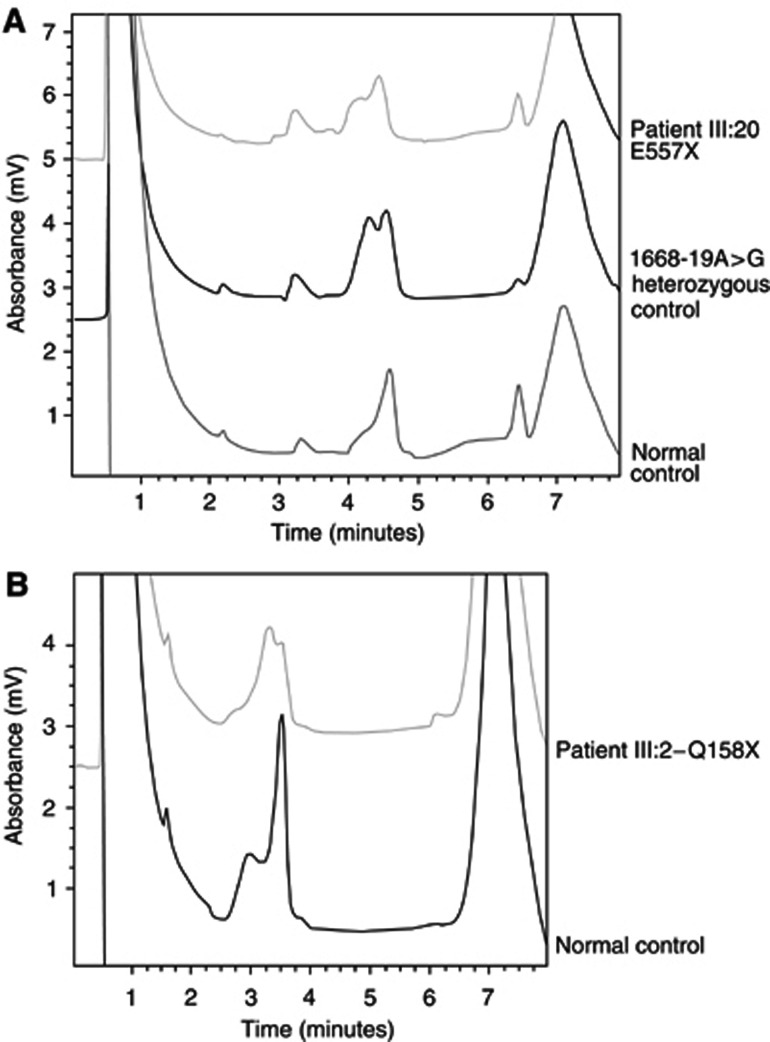
dHPLC analysis of two of the mutations identified: (**A**) E557X, (**B**) Q158X. Patient number refers to the pedigrees in Figures 1A and B, respectively.

**Figure 3:**
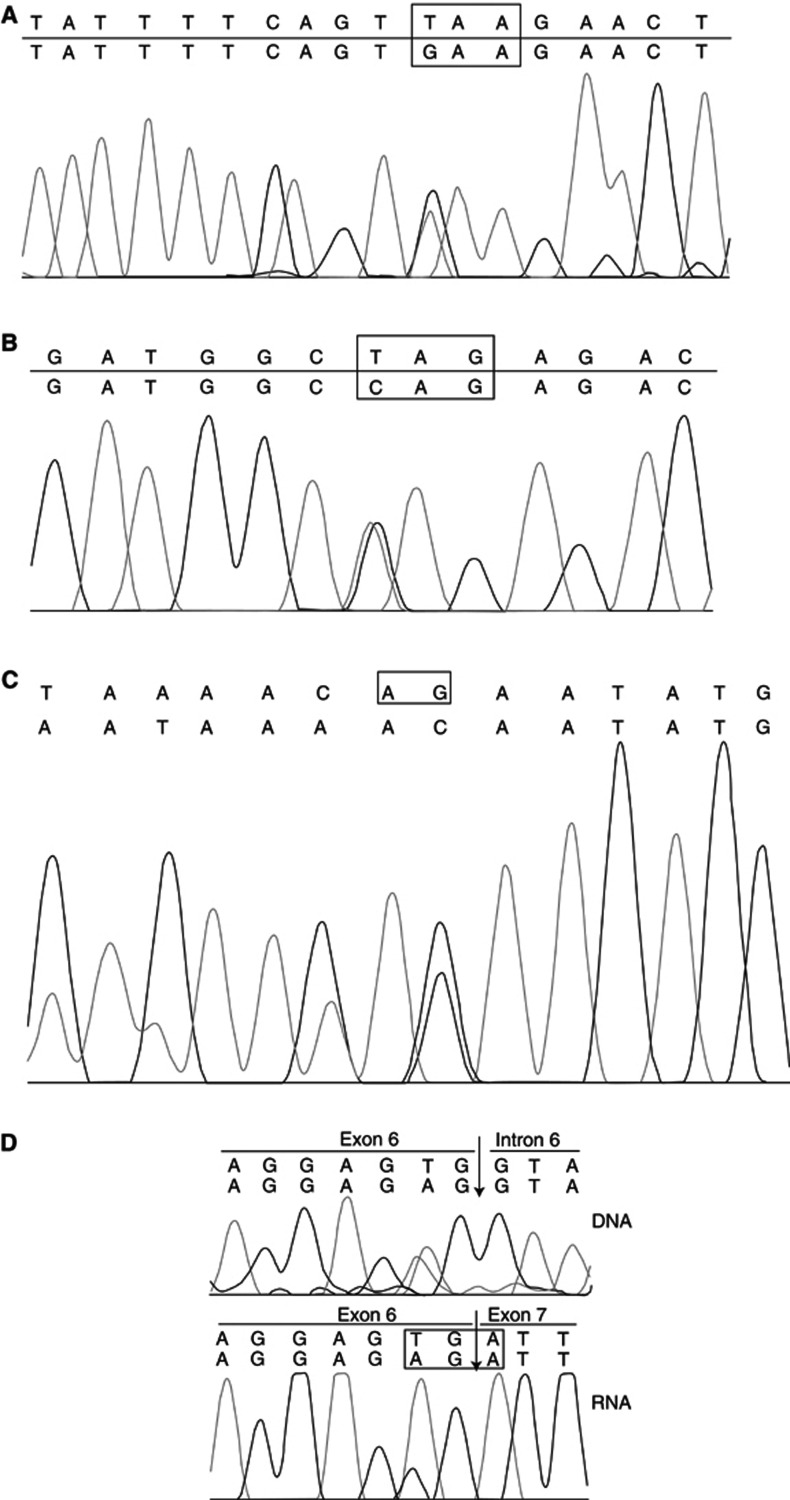
Chromatograms of novel mutations detected in this study. (**A**) E557X and (**B**) Q158X. The box indicates the mutated codon. (**C**) 1704_1705 delAG. The box indicates the deleted nucleotides. (**D**) R359X. Top panel=sequencing analysis of genomic DNA, the arrow indicates the 3′ splice junction. Bottom panel=sequencing analysis of cDNA, the box indicates the mutated codon.

In family 8344 (Figure 1B, Table 1), a mutation was suggested by dHPLC in the amplicon encompassing exon 3 of the *hMSH2* gene (Figure 2B). The mutation was shown by sequencing to be a C>T substitution of nt 472 of the cDNA (Figure 3B) and results in substitution of a glutamine at position 158 by a STOP codon. The mutation was identified in a patient who was diagnosed with CRC at the age of 38. His sister, who was also shown to carry the mutation developed a polyp of the large intestine at the age of 27. Their brother, who at the age of 37 is asymptomatic was found not to carry the family mutation.

In family 9663 (Figure 1C, Table 1), the proband (II:6, Figure 1C) was diagnosed with endometrial cancer at the age of 52 and CRC at 65. Her father (I:2, Figure 1C), brother (II:2, Figure 1C) and son (III:3, Figure 1C) also suffered from CRC while her niece (III:1, Figure 1C) died from endometrial cancer at the age of 34. Denaturing high-performance liquid chromatography analysis revealed a mutation in exon 11 of *hMSH2.* Sequencing showed this to be a 1704_1705delAG, which results in frameshift and creates a stop codon at 570 (Figure 3C). The mutation was also identified in the proband's son (III:3, Figure 1C), and one of her daughters (III:6, Figure 1C) who at the age of 44 is asymptomatic.

The patient from family 10107 (Figure 1D, Table 1) was first diagnosed with cancer of the ovaries at the age of 25, followed by endometrial cancer at 36, cancer of the urinary tract at 57 and CRC at 59. In addition, there were other cases of CRC, cancer of the eye, breast, endometrium, prostate and melanoma in the family. Sequencing of exon 6 of *hMSH2* revealed a G>T substitution at the last base of the exon (nt 1076). In order to confirm the effect of this mutation, that is, whether it results in a splicing defect or substitution of the amino acid by a stop codon, RT–PCR and sequencing was carried out. These showed that the mutation results in substitution of an arginine residue by a stop codon at 359 (Figure 3D). No other family members were available for analysis.

### Large genomic rearrangements

**Figure 4:**
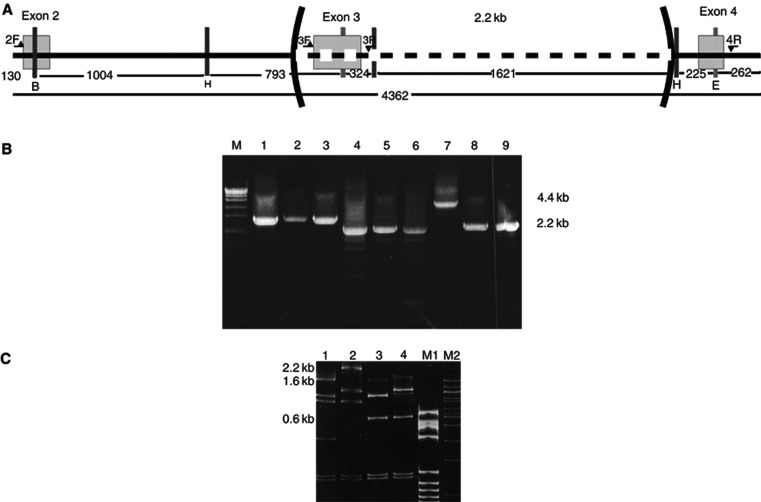
Characterisation of the 2.2 kb deletion identified in family 7562. (**A**) Schematic representation (not to scale) of the fragment of *hMSH2* gene amplified by long PCR showing the position of primers (horizontal arrows), exons (empty boxes), restriction endonuclease cleavage sites (vertical bars: H=*Hin*dIII, E=*Eco*RI, B=*Bgl*II) and the deletion. (**B**) Long-range PCR using combinations of primers (from **A**) spanning the repeat. M=molecular weight marker (VII, Boehringer Manheim, Germany), lanes 1–3 using primers 2F and 3R, 1=normal DNA, 2–3=patient DNA; lanes 4–6 using primers 3F and 4R, 4=normal DNA, 5–6=patient DNA; lanes 7–9 using primers 2F and 4R, 7=normal DNA, 8–9=patient DNA. (**C**) Restriction endonuclease digestion of PCR products from B with *Hin*dIII, *Eco*RI and *Bgl*II. The bands containing exon 3 (2.3 kb in lane 1 and 1.6 kb in lane 2) have disappeared and a new band 0.6 kb in length resistant to digestion with any of the three enzymes appears in the mutant sample (lanes 3 and 4). 1: normal control digested with *Eco*RI/*Hin*dIII/*Bgl*II. 2: normal control digested with *Eco*RI/*Hin*dIII. 3: patient DNA digested with *Eco*RI/*Hin*dIII/*Bgl*II. 4: patient DNA digested with *Eco*RI/*Hind*III. Lane M1: molecular weight marker V (Boehringer Manheim, Germany). Lane M2: Molecular weight marker 100 bp (New England Biolabs).

In family 7562 (Figure 1E, Table 1), MLPA analysis for screening of large genomic rearrangements revealed a deletion comprising exon 3 of the *hMSH2* gene. Long-range PCR using primers located in introns 2 and 4 (Figure 4) confirmed the deletion, which was found to be 2.2 kb long (Figure 4B). Subsequent fine mapping of the deletion breakpoints using a mixture of restriction endonucleases (Figure 4C) allowed the design of a new primer suitable for sequencing of the breakpoints. These were shown to be located in two *Alu* repeats in introns 2 (*Alu*Sg) and 3 (*Alu*Sx), respectively, sharing 78% homology. The deletion was flanked by 21 bases of complete homology. It is an in-frame deletion and results in the absence of 93 aa residues from the resulting protein product. The proband was a female patient who was diagnosed with CRC at the age of 35. The patient's father had died from CRC, her sister was diagnosed with endometrial cancer at the age of 45 and her brother developed CRC at the age of 42. The mutation was later identified in the siblings of the patient and in her daughter who at the age of 29 years is an asymptomatic carrier. MSI analysis of tumour DNA from the proband was found to be negative.

### Families with known mutations

In family 5838 (Figure 1F, Table 1), 676C>T was identified by sequencing in exon 8 of the *hMLH1* gene. The mutation results in the substitution of an arginine codon at position 226 by a stop codon and has previously been described by others (Moslein *et al*, 1996; Wehner *et al*, 1997). It was originally identified in a patient who had been diagnosed with CRC at the age of 49 (IV:1 in Figure 1F) who came from a family with multiple cases of CRC, two cases of endometrial cancer, both of whom carry the mutation, in addition to cancer of the larynx and the stomach.

In family 8902 (Figure 1G, Table 1), there were three cases of CRC, three cases of gynaecological cancers and one individual for whom no information of cancer type was available. Denaturing high-performance liquid chromatography analysis identified a mutation in exon 13 of the *hMSH2* gene, whose sequencing was shown to be C>T mutation at nucleotide 2131, resulting in substitution of the arginine at codon 711 by a stop codon. This mutation has been previously described by others (Kurzawski *et al*, 2002).

## DISCUSSION

In this study we present mutation analysis of the *hMLH1* and *hMSH2* genes implicated in the HNPCC syndrome in a cohort of nine Greek families at high risk of having HNPCC. Two of the families do not strictly conform to the Amsterdam II criteria. Family 8344 (Figure 1B) meets the Bethesda criteria since cancer was diagnosed in the proband at the age of 38. Family 9659 (Figure 1I) was included in the analysis due to the number of affected members (four individuals with CRC and one case of endometrial cancer). The pathogenic mutation was identified in seven out of nine (78%) families, including also one of the non-Amsterdam families (8344). This percentage compares well with other studies (Caluseriu *et al*, 2001; Wagner *et al*, 2003). However, it should be stressed that the accuracy and reliability of family history provided by patients and their relatives may not always be accurate and may therefore misguide researchers in the assignment of a family into a particular risk status group.

Interestingly, the majority of mutations (five out of seven, i.e. 77.7%) identified in this study are located in the *hMSH2* gene. Although our sample group is quite small, this finding is in contrast with previous studies where the majority of pathogenic mutations associated with HNPCC have been identified in *hMLH1* (Peltomäki and Vasen, 1997; Wagner *et al*, 2003). Furthermore, four of the six single-point substitutions/small deletions are novel mutations, not previously described in the ICG-HNPCC mutation database (http://www.nfdht.nl). All single-point substitutions/small deletions identified in this study are predicted to introduce premature stop codons in the gene sequence, therefore, resulting in truncated protein products. One of the novel mutations occurs at the last base of exon 6 of the *hMSH2* gene. The effect of this mutation on the protein was further investigated using RT–PCR and sequencing, showing that the mutation results in substitution of an arginine residue by a stop codon at 359. This, in addition to, segregation of these novel mutations with the disease phenotype in the majority of cases confirms their pathogenic nature. One individual in each of the families, 7562, 8902 and 9663 in Table 1, was found to be an asymptomatic carrier. However, all three individuals are relatively young, 29, 14 and 44, respectively, while the average age at onset of the disease is quoted as being ∼45 years of age according to the Amsterdam II criteria (Vasen *et al*, 1999). Furthermore, the lifetime risk of developing CRC in carriers of an MMR gene mutation is ∼80% (Vasen *et al*, 1996; Aarnio *et al*, 1999).

The mutations identified in this and other studies are scattered throughout the two genes, with no obvious phenotype–genotype correlation. This, in addition to identification of both single-point substitutions and small deletions, as well as a 2.2 kb deletion, necessitates screening of the entire genes both for single-point mutations and small insertions/deletions as well as large genomic rearrangements. Even in this small cohort of patients genomic rearrangements account for 14.2% of identified mutations. Our results, therefore, indicate the importance of applying a variety of molecular biology techniques in order to identify inactivating mutations in high-risk patients.

Another interesting point emerging from our study of Greek HNPCC families is that a unique mutation was identified in each kindred. This is in contrast to other populations such as the Finnish where two mutations account for >50% of the HNPCC families (Nystrom-Lahti *et al*, 1995).

Clinical diagnosis of HNPCC is not always easy as there is no clear phenotype associated with the disorder as is the case with other cancer predisposition syndromes such as familial adenomatous polyposis (Mihalatos *et al*, 2003b). Besides the spectrum of cancers characteristic of HNPCC, MSI is considered as one of the hallmark diagnostic features of HNPCC-related cancers (Boland *et al*, 1998). However, in this study, a pathogenic mutation was identified in the *hMSH2* gene in a patient whose CRC tumour was found to be microsatelite stable (family 7562). This could be explained by the absence of enough cancerous material in the sample analysed. However, if MSI analysis were a strict criterion in the inclusion of patients for genetic analysis, this patient would have been excluded. This finding stresses the need for use of a combination of criteria in the selection of patients suitable for mutation screening.

The reason for the interfamilial variability in the phenotypic manifestation of HNPCC is still not clear. However, use of DNA microarray analysis should soon shed some light as to the genetic factors that may act as modifiers in the disease phenotype predicted by the detection of germline mutations in MMR genes.

In summary, we have analysed nine families at high risk of carrying mutations in one of the MMR genes. The pathogenic mutation was identified in seven of the families. Three of the mutations identified are novel single base nonsense mutations, while a novel small deletion also resulting in premature termination has been identified. Finally, two previously described nonsense mutations and a 2.2 kb deletion in *hMSH2* were also identified. The results presented here provide the spectrum of mutations responsible for HNPCC for the first time in the Greek population, underscoring the differences observed in different geographic populations.

## Figures and Tables

**Table 1:** Variants identified in the *hMLH1* and *hMSH2* genes during this study

**Gene**	**Family ID**	**Risk status^a^**	**MSI**	**Exon**	**Nucleotide**	**Effect**	**Types of cancer**
*Mutation*							
*hMLH1*	**3335**	Amsterdam	4/5	**15**	**1669G>T** b	**E557X**	CRC^c^, melanoma, liver, TCC^d^, endometrium, lung
	5838	Amsterdam	NT	8	676C>T	R226X	CRC^c^, endometrium, larynx, stomach
							
*hMSH2*	7562	Amsterdam	0/5	3	Del^e^ 2.2 kb	Protein missing aa 123–215	CRC^c^, endometrium
	**8344**	Bethesda	4/5	**3**	**472C>T** b	**Q158X**	CRC^c^
	8902	Amsterdam	5/5	**13**	2131C>T	R711X	CRC^c^, ovaries, endometrium
	**9663**	Amsterdam	NT	**11**	**1704_1705del**^e^ **AG**^b^	**Fs**^f^ **resulting in STOP at codon 570**	CRC^c^, endometrium
	**10107**	Amsterdam	NT	**6**	**1076G>T** b	**R359X**	CRC^c^, ovary, endometrium, urinary tract, eye, prostate, melanoma, breast
							
**Gene**	**Exon**	**Codon**	**Nucleotide change**	**Consequences**	**No. of families**		
*Benign polymorphisms*							
*hMLH1*	**IVS 4**	**309−30** b	**c>a**	—	2		
	Exon 8	219	655A>G	I219V	5		
	**Exon 12**	**464** b	**464T>C**	**P464P**			
	**IVS 12**	**1408−54** b	**c>t**	**—**	2		
	IVS 13	1558−13	g>a	—	2		
	IVS 14	1668−19	a>g		6		
							
*hMSH2*	IVS 1	211+9	c>g	—	1		
	**Exon 4**	**224** b	**669C>T**	**L224L**	2		
	IVS 9	1511−9	a>t	—	1		
	IVS 10	1661+13	g>a	—	2		
	IVS 12	2006−6	t>c	—	2		

a Risk status based on the Amsterdam II (Vasen *et al*, 1999) and Bethesda (Rodrigues – Bigas *et al*, 1997) criteria.

b Novel variations identified in this study (marked in bold).

c CRC=colorectal cancer.

d TCC=transitional cell carcinoma.

e Del=deletion.

f Fs=frame shift mutation.
